# Supplementary material for: Human Placental NADPH Oxidase Mediates sFlt-1 and PlGF Secretion in Early Pregnancy: Exploration of the TGF-β1/p38 MAPK Pathways
Source: Antioxidants (Basel). 2021 Feb 12;10(2):281. doi: 10.3390/antiox10020281 (PMC7918586; doi:10.3390/antiox10020281)
Supplement: Supplementary file 1 [file antioxidants-10-00281-s001.pdf]

Supplementary Material S1: p38/phospho-p38 Western blots: kinetic

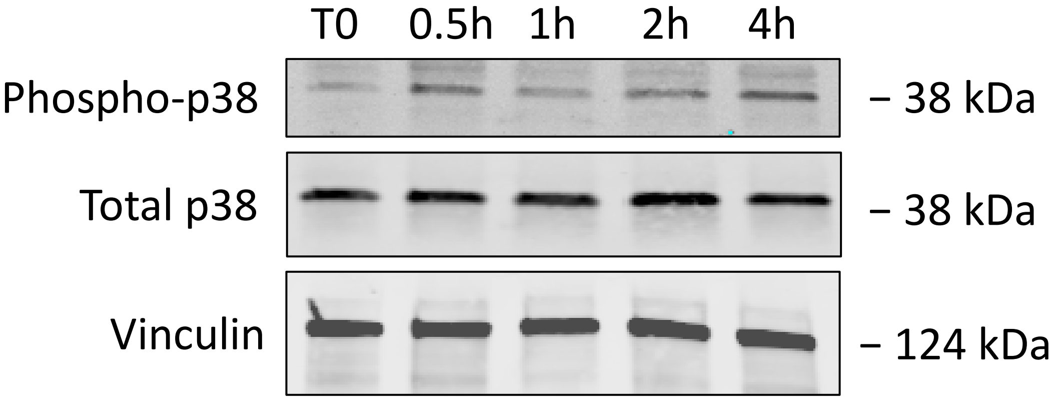

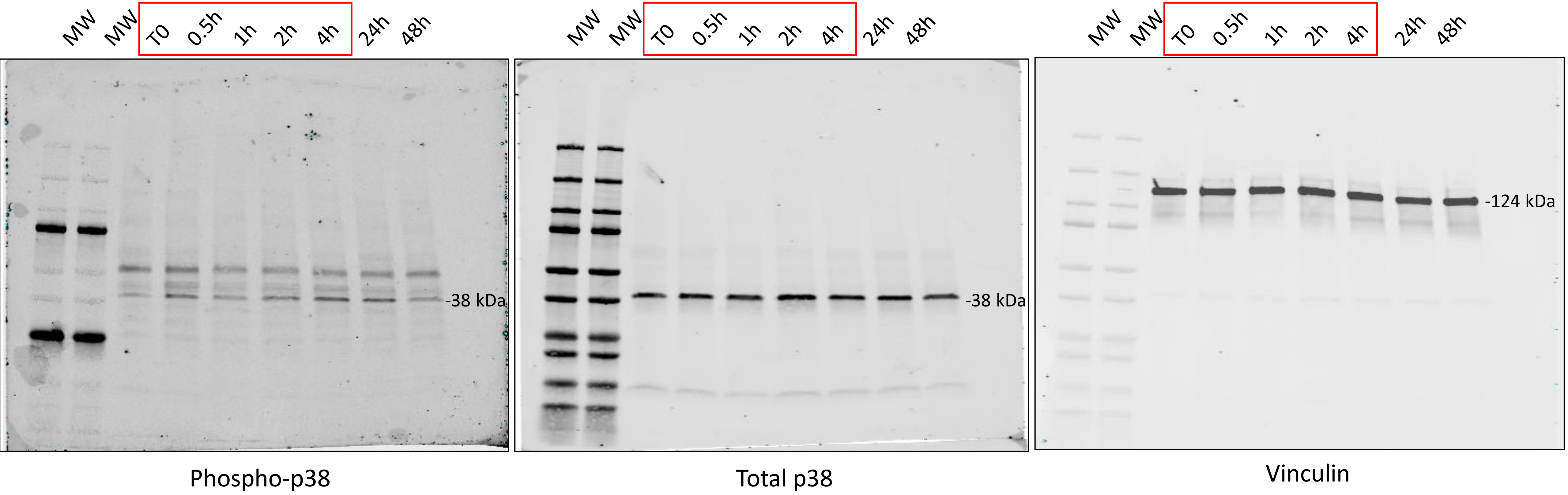

Abbreviations: kDa: kilodalton, MW: molecular weight, phospho-p38: phosphorylated p38 MAPK protein, total p38: total p38 MAPK protein, T0: time zero

Supplementary Material S2: p38/phospho-p38 Western blots

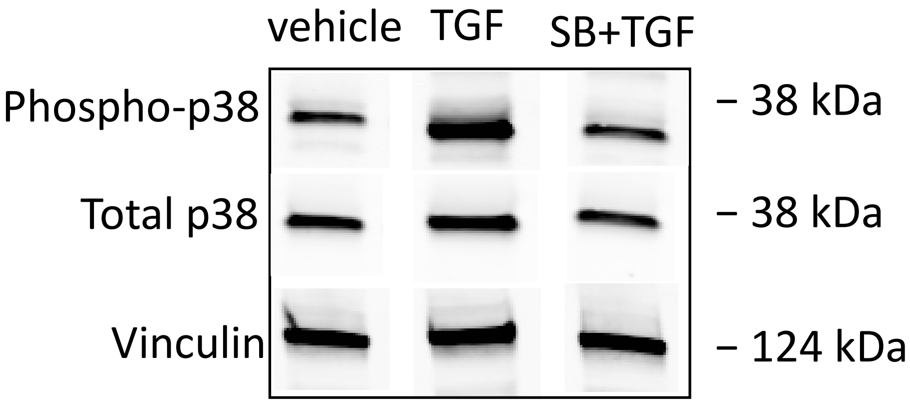

SB 10: SB203580 10 µg/mL
SB 20: SB203580 20 µg/mL
SB10+TGF: SB203580 10 µg/mL + TGF-β1 10 ng/mL
SB20+TGF: SB203580 10 µg/mL + TGF-β1 20 ng/mL

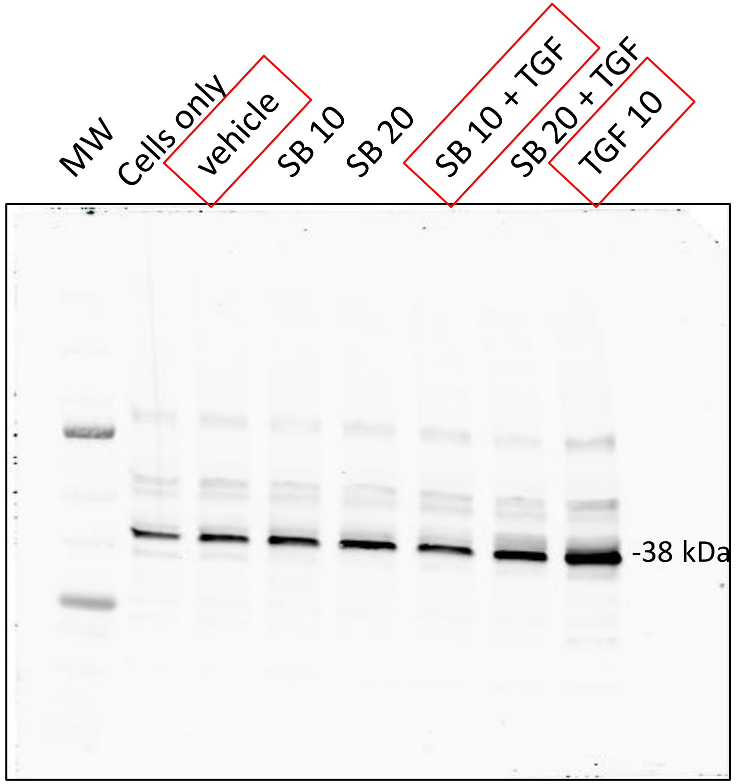

Phospho-p38

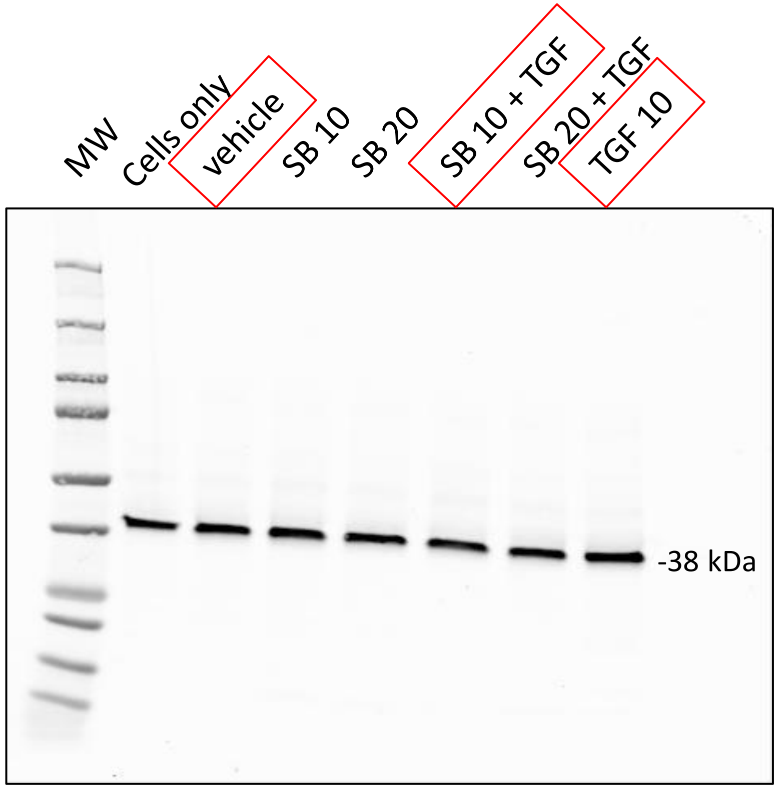

Total p38

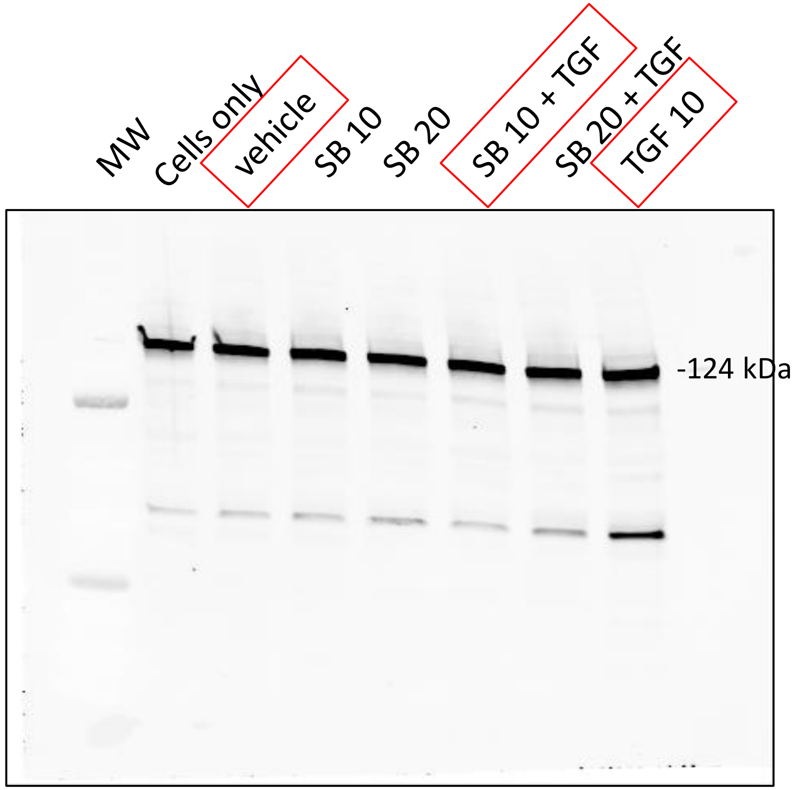

Vinculin

Abbreviations: kDa: kilodalton, MW: molecular weight, phospho-p38: phosphorylated p38 MAPK protein, SB: SB203580, TGF-β1: Transforming Growth Factor beta 1, total p38: total p38 MAPK protein

Supplementary Material S3: SMAD2/phospho-SMAD2 Western blots: kinetic

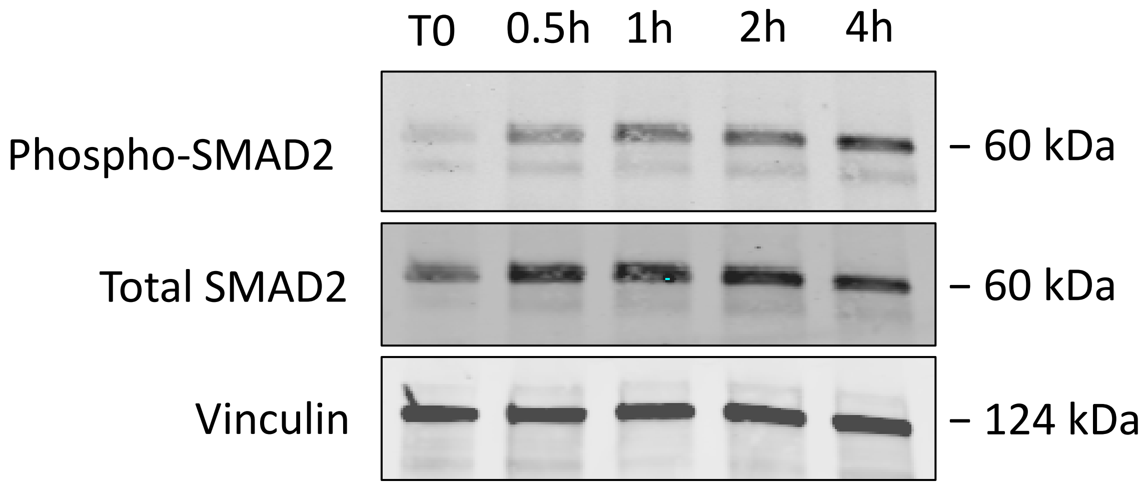

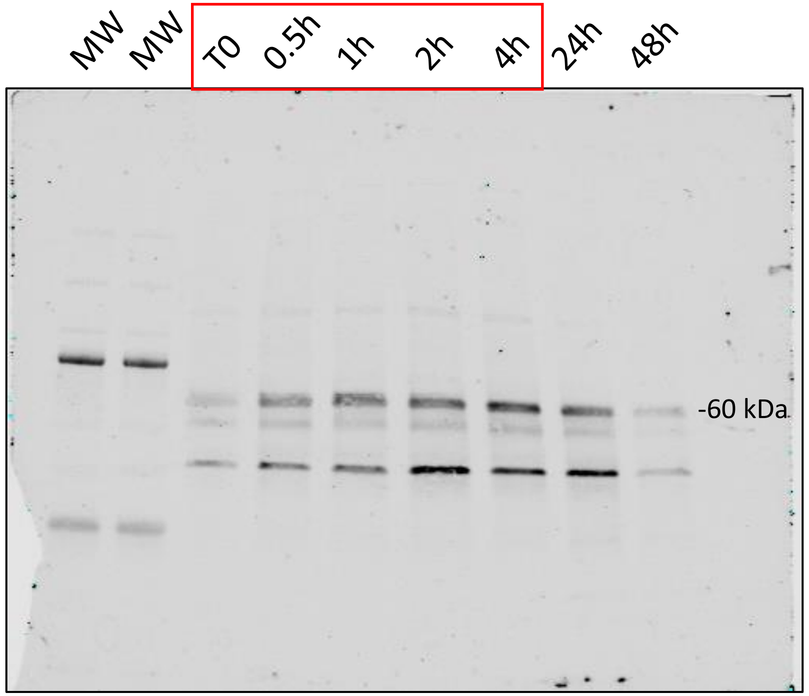

Phospho SMAD2

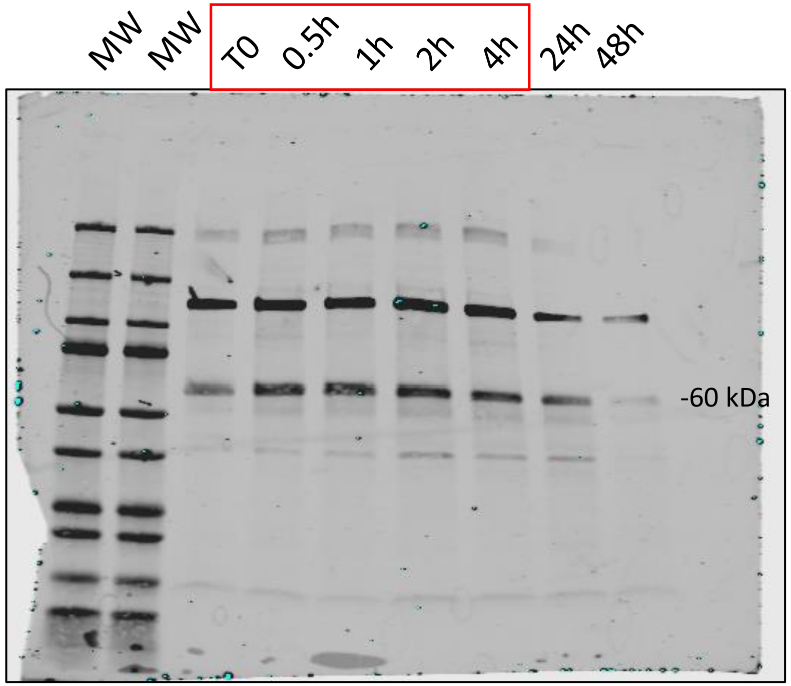

Total SMAD2

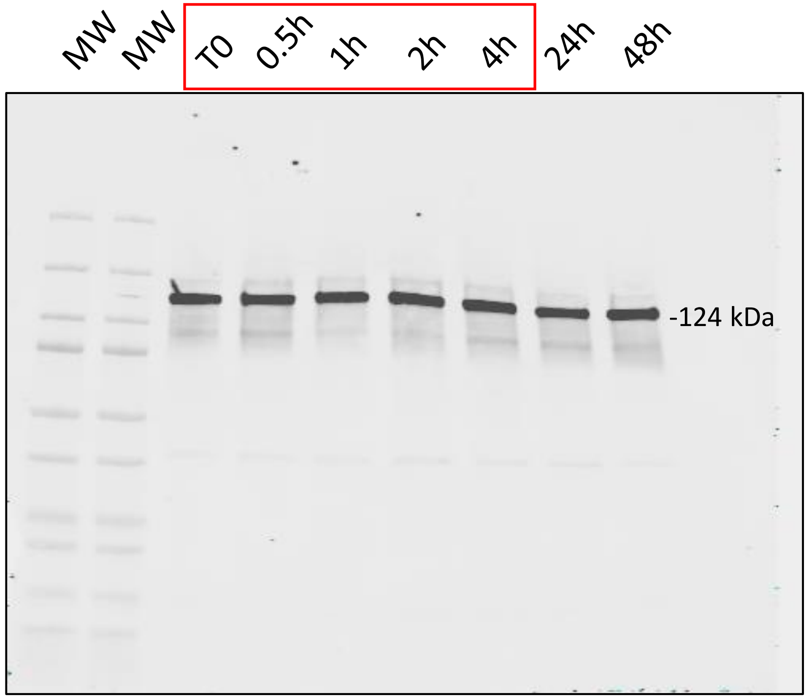

Vinculin

Abbreviations: kDa: kilodalton, MW: molecular weight, phospho-SMAD2: phosphorylated SMAD2 protein, SMAD2: Mothers Against Decapentaplegic homolog 2, T0: time zero

Supplementary Material S4: SMAD2/phospho-SMAD2 Western blots

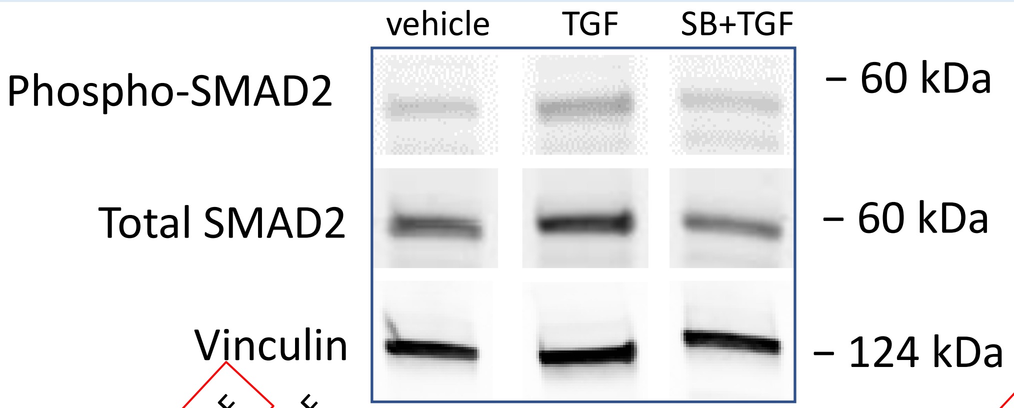

SB 10: SB203580 10 µg/mL
SB 20: SB203580 20 µg/mL
SB10+TGF: SB203580 10 µg/mL + TGF-β1 10 ng/mL
SB20+TGF: SB203580 10 µg/mL + TGF-β1 20 ng/mL

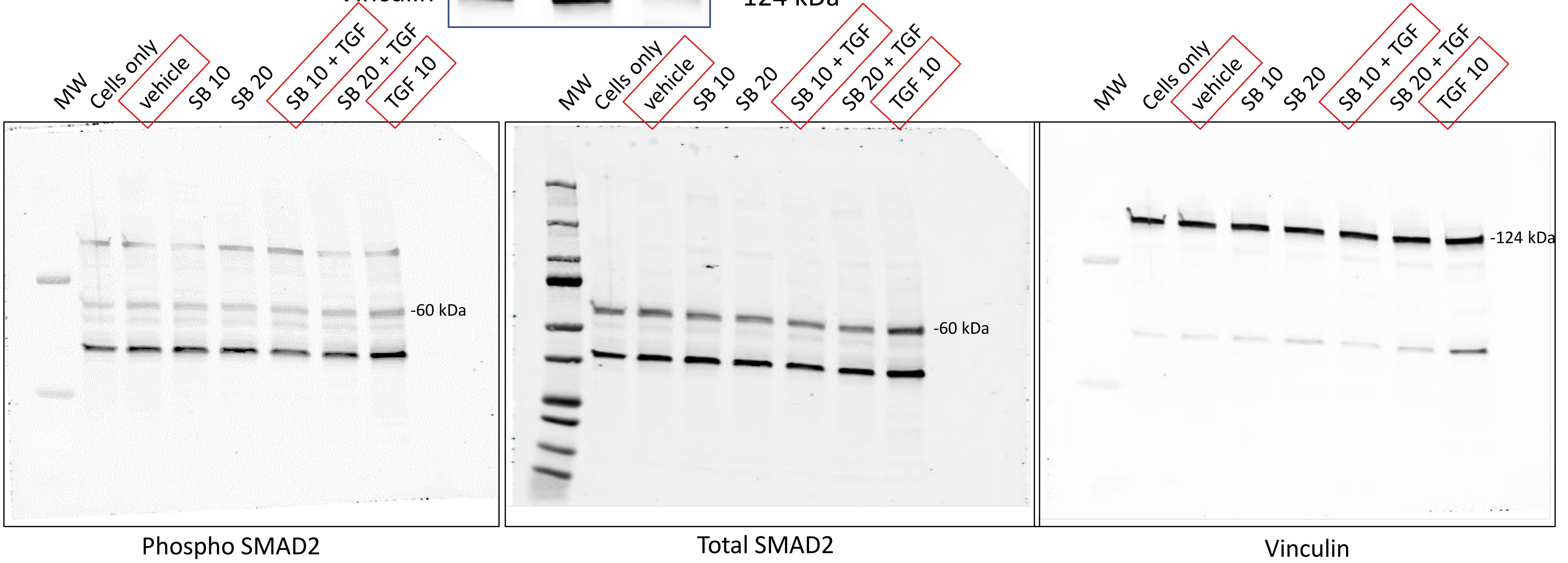

Abbreviations: kDa: kilodalton, MW: molecular weight, SB: SB203580, phospho-SMAD2: phosphorylated SMAD2 protein, SMAD2: Mothers Against Decapentaplegic homolog 2, TGF-β1: Transforming Growth Factor beta 1 , T0: time zero
